# Why Do People Want Dogs? A Mixed-Methods Study of Motivations for Dog Acquisition in the United Kingdom

**DOI:** 10.3389/fvets.2022.877950

**Published:** 2022-05-10

**Authors:** Katrina E. Holland, Rebecca Mead, Rachel A. Casey, Melissa M. Upjohn, Robert M. Christley

**Affiliations:** Canine Behaviour and Research, Dogs Trust, London, United Kingdom

**Keywords:** dogs, dog acquisition, human-animal relationships, mixed-methods, qualitative

## Abstract

With an estimated 12. 5 million dogs in the UK alone, many people acquire a dog at some point during their lives. However, there are gaps in understanding about why UK owners decide to get dogs. Using a mixed-methods convergent design, this study identified the reasoning behind dog acquisition in a sample of UK current and prospective owners. An online survey of current (*n* = 8,050) and potential (*n* = 2,884) dog owners collected quantitative and qualitative data. Current owners were asked about the acquisition of their most recently acquired dog, whilst potential owners were asked about their dog ownership aspirations. Additional qualitative data were collected through semi-structured interviews with current (*n* = 166) and potential (*n* = 10) dog owners. Interviews focused on the factors that affected why and how people acquire dogs. Of survey responses, companionship for the respondent was the most common reason for wanting to get a dog, reported by 79.4 and 87.8% of current and potential owners, respectively. Facilitating exercise was reported as a reason for wanting to get a dog by 48.2 and 69.7% of current and potential owners, respectively. There were significant differences between current and potential owners in their likelihood of reporting pre-defined reasons, factors and influences involved in their decision to get a dog. Compared to current owners, potential owners were significantly more likely to report being motivated by most of the survey response options offered (including companionship for themselves or other adults in the household, helping a dog in need, lifestyle changes and previous experiences of meeting dogs), suggesting that current ownership status may affect experience and/or reporting expectations around dog ownership. Reflexive thematic analysis of qualitative data confirmed the importance of these motivations and identified additional reasons and factors that drive dog acquisition. These were organized into three overarching themes: *Self-Related Motivation, Social-Based Motivation*, and *Dog-Related Positive Affect-Based Motivation*. These findings provide insights into owners' expectations of ownership which may inform the development of interventions to support potential owners' decision-making around acquisition to maximize both dog and human welfare.

## Introduction

The domestic dog (*Canis familiaris*) is among the most common species of pet animal in many countries, including the UK where there are an estimated 12.5 million dogs (1) and 26% of adults are understood to own one (2).^1^ Although many owners report feeling highly satisfied with their relationship with their dog (4), it is estimated that between 90,000 and 130,000 dogs are relinquished to UK animal welfare organizations annually (5, 6). Previous evidence suggests that one risk factor for relinquishment is owners' expectations of the roles pets will play (7). Therefore, understanding the reasons people acquire dogs is important for animal welfare organizations.

Whilst dogs were historically kept for various practical purposes, including guarding and hunting, privately owned dogs across Western societies today are largely considered to be pets. This category of animals–as pets–contrasts with dogs kept for specific working purposes, such as those used by police or military forces. In practice, of course, these two categories may overlap, for instance in the case of guide dogs, where the dog performs a working role but is also considered a companion by their owner, at least some of the time (8). The present study concerns privately owned–and thus, broadly speaking–pet dogs.

As pets are considered to lack an economic or practical function (9), the popularity of pet ownership presents a paradox from an evolutionary perspective (10). Although some people continue to acquire dogs to fulfill a working role (e.g., security, herding or assistance) (11), ample evidence suggests the main reason for pet acquisition is companionship for the owner (11–14). Another common reason for getting a dog is companionship for others in the household (including other humans–children and adults–and other dogs) (11). Beyond companionship, perceived health-related benefits, including physical exercise and mental health improvements, are also commonly cited reasons for dog acquisition (11, 15, 16). Evidence suggests that the anticipated benefits and roles of dogs may be associated with ownership experience, with current owners and people with previous ownership experience having increased likelihood of anticipating physical, mental and psychosocial benefits compared with people who have never owned a dog (15). Previous evidence illustrates the types of relationships formed between humans and dogs may help explain why so many people choose to live with a dog. Beyond simply providing care for a pet, many owners form a close relationship with them, perceiving them as friends (17), family members (17–20) and even as proxy children (17, 21, 22).

A study by Beverland et al. (23) suggests that it may be possible to differentiate between pet owners who are intrinsically motivated in their ownership and others who are driven by extrinsic motivations. According to this study, intrinsically motivated owners value their pets for the sake of the individual animal and seek to achieve goals that are innately satisfying. This type of ownership contrasts with extrinsically-motivated ownership, in which owners are concerned with the self-relevant benefits they can gain from pet ownership, including interpersonal interactions facilitated by dogs (21) and the status or acknowledgment from others when dogs function as extensions of the owner's self, (17, 21). Other self-related motivations for ownership may include emotional and social support received from dogs and their role in keeping the owner active (24). Beverland et al.'s findings also highlight how different motivations for acquisition may affect how owners treat their dogs, with extrinsically-motivated owners more likely to treat their dogs as objects for human pleasure rather than individuals with needs to consider (23).

Despite the sustained popularity of dogs in the UK (25), to date, little research into owners' reasons and motivations for acquiring dogs has been conducted in this country. One exception is a recent study by Packer et al. (11) which provided insights into reasons for puppy acquisition amongst UK owners. However, given this study's exclusive focus on the purchase of puppies, there remains an evidence gap on motivations for acquisition of dogs across their lifespan in the UK. This study aimed to address the limitations of previous studies by applying a mixed-methods approach to identify the motivations for dog acquisition (inclusive of puppies and adults) amongst a sample of UK dog owners.

## Materials and Methods

### Ethics Statement

Ethical approval for this study was granted by the Dogs Trust Ethical Review Board (reference numbers: ERB018 and ERB019). Informed consent was obtained from all participants prior to participation in either the survey or interview aspect of this study.

### Study Design

**Figure 1:**
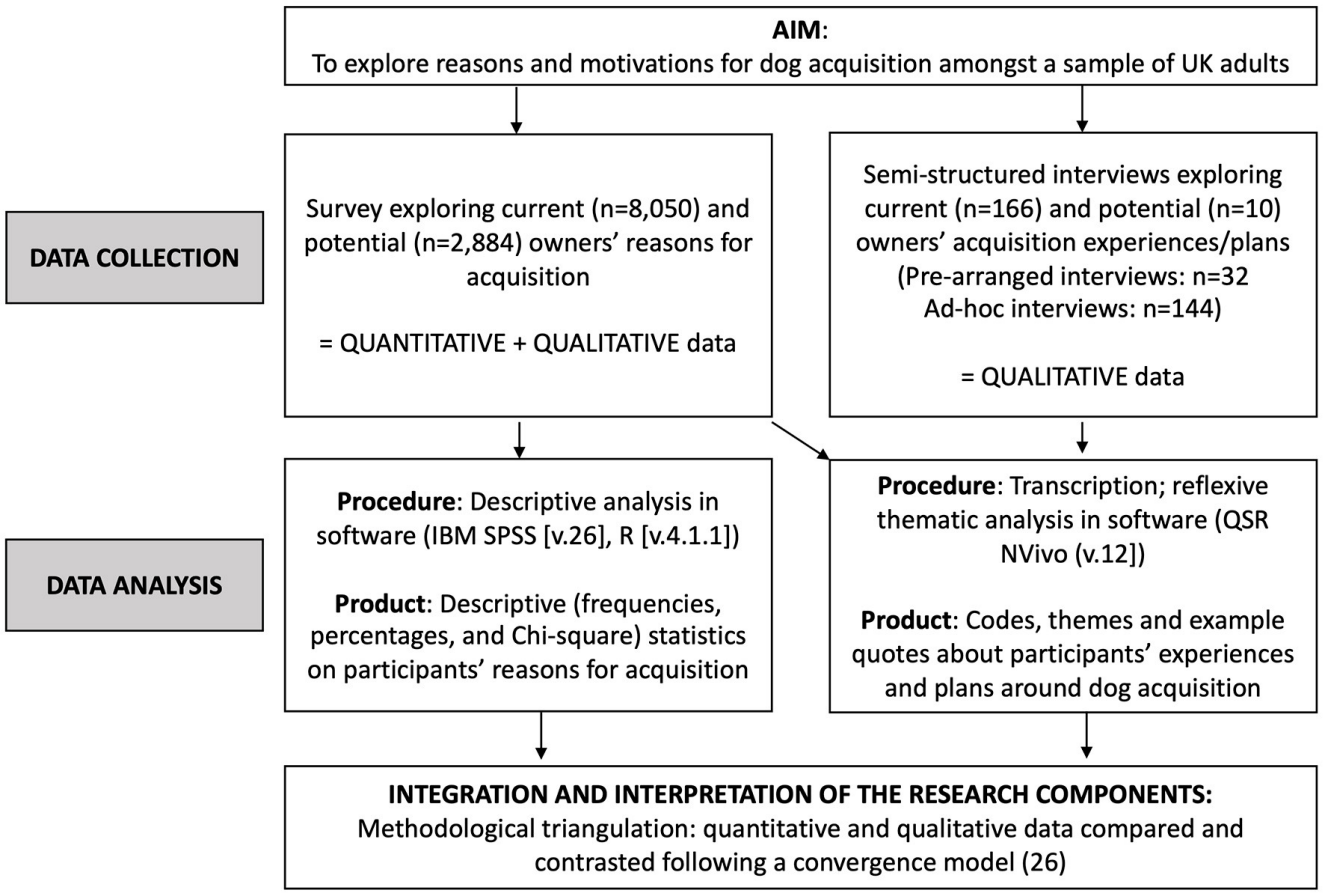
Procedural diagram of the mixed-methods study design.

This study used a convergent mixed-methods study design to collect complementary data (Figure 1). Data were collected largely in parallel, analyzed independently and interpreted together in a comparative and contrasting way (26).

### Data Collection

#### Survey: Design and Content

A self-completion online survey on SmartSurvey^TM^ (www.smartsurvey.com) was designed to collect data about the pre-acquisition and acquisition motivations, behaviors and experiences of current and potential dog owners in the UK. Informed by a review of current literature (27), survey questions were developed amongst the authors. Prior to launch, the survey was piloted to ensure ease of comprehension and test survey logic. Firstly, 12 members of Dogs Trust staff completed the survey and provided feedback to this study's authors. Additionally, the survey was piloted with 110 (current or potential) dog owners, recruited *via* two promotional posts on the Facebook page of “Generation Pup” (https://www.facebook.com/generationpup/). Generation Pup is an ongoing longitudinal cohort study of the health, welfare, and behavior of dogs (28). Following this pilot phase, minor changes were made to the survey's logic to optimize respondent's ease of completion.

All respondents were asked if they owned at least one dog at the time of survey completion, as well as whether they were considering acquiring a/another dog in the future. Therefore, the survey collected data from two types of owners: “current” and “potential” owners. Current owners who owned multiple dogs were asked to respond based on the acquisition of their most recently acquired dog. If respondents had acquired more than one dog at the same time, they were asked to answer for the dog whose name comes first alphabetically. Survey questions relevant to this study focused on reasons and motivations for dog acquisition. Respondents were asked about their reasons for wanting to get a dog via both open-ended and fixed-choice multiple-choice questions. We also collected self-reported demographic information about respondents and, where applicable, their (most recently acquired) dog. The time required to complete the survey was ~20 min, however the number of questions per participant (and thus the completion time) varied depending on their dog ownership status (i.e., a current owner who was also considering getting another dog was asked more questions than a potential owner who did not own a dog at survey completion). Original survey questions relevant to this study can be found in the Supplementary Material for this article. More detail about the specific questions relevant to reasons and motivations for acquisition, asked to current and potential owners respectively, is provided below.

##### Survey Design and Content: Current Owners

Respondents who owned a dog at the time of survey completion (*n* = 8,050) were first asked about their reasons for wanting to get a dog via the following open-ended question: “Can you describe why you wanted to have a dog?” This open-ended question was included to avoid bias by suggesting responses to respondents. Later in the survey, several multiple-choice questions with fixed-choice response options (multiple answers possible) were included to collect quantitative data regarding reasons, contributing factors and influences around the acquisition of their current dog. In addition to fixed-choice response options for these questions, the free-text response option “Other, (please specify)” was included, to gather novel insights and additional information. This generated additional qualitative data.

Current owners were asked if they were considering acquiring another dog soon. Those who reported that they were (*n* = 2,205), were asked to describe why they wanted to acquire another dog via the open-ended question: “Can you describe why you want to have another dog?”

##### Survey Design and Content: Potential Owners

The survey was also open to people who did not own a dog at the time of survey completion, but who responded that they were seriously considering acquiring one soon (i.e., “potential owners”). The term “seriously considering” was not defined within the survey but respondents who did not currently own a dog were categorized as potential owners if they selected either “Yes: I am seriously considering getting a dog in the next 6 months but I'm not actively looking at the moment” or “Yes: I am seriously considering getting a new dog but this probably won't be in the next six months” to describe their future ownership plans. As with the questions presented to current owners, potential owners (*n* = 2,884) were first asked about their reasons for wanting to get a dog via the following open-ended question: “Can you describe why you want to have a dog?” Later in the survey, several multiple-choice questions with fixed-choice response options (multiple answers possible) were included to collect quantitative data regarding reasons, contributing factors and influences around dog acquisition. In addition to fixed-choice response options for these questions, the free-text response option “Other, (please specify)” was included.

##### Survey: Participant Recruitment

The survey was promoted through Dogs Trust *via* the charity's website, social media pages, contact center, rehoming centers, retail shops and Dog School dog training classes, and through correspondence with supporters (e.g., Dogs Trust's E-Newsletter and WAG magazine). The survey was open to respondents to complete for 3 months (25 September 2019–31 December 2019), with no payment or incentive offered for participation. Participants were required to be aged 18 years and over and living in the UK.

#### Interviews

Two types of interviews were conducted: (a) pre-arranged and (b) *ad-hoc* interviews. All interview participants were required to be 18 years or older to take part. Both types of interviews followed a semi-structured guide (see Supplementary Material) which covered broad topics focused on various aspects of the acquisition process, including motivations for the acquisition. To test the appropriateness of interview questions, interviewing style and approach, interviews were piloted in a similar manner to the survey. First, we conducted pilot interviews with 12 Dogs Trust staff members. Additional pilot interviews were conducted with five respondents to the pilot survey. As part of the pilot phase, individual and group interviews (with 2–3 participants) were trialed. Individual interviews were identified as the best approach to achieve the objectives of our research questions, which focused on gathering information about the experiences or plans of individuals. However, the interview guide was not amended following the pilot phase and interview data from the pilot phase contributed to the final analysis. Each interview was conducted by one of three authors (K.E.H., R.M., or R.M.C.).

##### Pre-arranged Interviews

Semi-structured interviews were conducted with current (*n* = 24) and potential (*n* = 8) UK dog owners between April 2019 and March 2020. Some of the current owners (*n* = 3) were also considering getting another dog at some point in the future. Many pre-arranged interviewees (*n* = 20) had completed the survey and had agreed to be contacted about further research opportunities, whilst others (*n* = 12) were Dogs Trust staff. Except for one interviewee–who participated in two follow-up interviews following the acquisition of their dog–participants were interviewed once each. Pre-arranged interviews were conducted either remotely, e.g., *via* telephone, (*n* = 22) or face-to-face (*n* = 10). Participants were not asked to provide demographic information. Interviews lasted between 17 and 60 min in length (mean = 33 min). With participants' consent, all pre-arranged interviews were audio recorded using a Dictaphone. Recordings were transcribed “intelligent verbatim” (i.e., false starts and filler words, such as “um” and “err” were omitted).

##### Ad-hoc Interviews

To gather data from a broader range of dog owners, interviews were also conducted at 23 Dogs Trust community events across the UK between May and December 2019. These free events allowed owners to obtain advice on topics including diet, exercise, and enrichment. A free microchipping service was also offered, and qualified veterinary nurses (excluding Northern Ireland events) provided free basic health checks. The locations for these events were determined using findings from Dogs Trust Stray Dog Survey data (29), and discussions with community partners (e.g., dog wardens and housing association staff) about local hotspots for dog-related issues and areas of deprivation. Participants were approached whilst waiting to speak to the event staff, or after the member of staff had attended to them (and their dog[s]). The nature and purpose of the interview was explained by the researcher and participants were invited to take part on-the-spot. *Ad-hoc* interviews were conducted with 142 current owners or carers (or sets of owners, where a dog was accompanied by more than one co-owner) and 2 potential owners. Participants were not asked to provide demographic information. With consent, 44.4% (*n* = 64/144) of *ad-hoc* interviews were audio recorded using a Dictaphone and lasted between 2 and 26 min in length (mean = 11 min). Recordings were transcribed in the same way as pre-arranged interviews. For interviews where participants did not give consent for audio recording, or where events were too noisy to enable clear audio recordings, the researcher made handwritten notes of responses that were later typed for analysis.

### Data Analysis

#### Quantitative Data Analysis

Following initial cleaning of data in Microsoft Excel and IBM SPSS (v.26), responses to relevant closed-ended survey questions were summarized with descriptive statistics (frequency and percentage) using IBM SPSS (v.26) and R (v.4.1.2) (30). Chi-square tests were used to compare responses given by two groups of respondents (i.e., grouped according to current ownership status) regarding reported reasons, influences, and factors involved in the decision to get a dog. Survey respondents were asked to provide the first half of their postcode; this was used to assign respondents to one of the four countries of the UK to assess the representativeness of the study sample.

#### Qualitative Data Analysis

Interview transcripts and relevant survey free-text responses were imported into NVivo (v.12, QSR). The data were analyzed using a reflexive thematic analysis (31) based on Braun and Clarke's six-phase process (32), in order to identify key themes and patterns occurring in responses. Three authors (KH, RM and RCh) familiarized themselves with the data, through listening to interview recordings and reading transcripts and free-text responses. The data were then first coded following an inductive approach (i.e., content driven and informed by the data), without using a pre-existing coding frame. These initial codes were refined through further analysis, reading and discussion in an iterative process through which new codes were introduced to capture the meaning of groups of initial codes. Subsequently, codes reflecting different aspects of reasoning behind dog acquisition were grouped into initial themes. A subsequent review of the themes included taking a deductive approach, as we drew on overarching themes developed in previous literature (16) to help organize our coding around patterns of shared meaning. The composition of the final themes was collaboratively established by the three authors involved in the analysis (KH, RM and RCh).

As is standard in most qualitative research, where the aim is to explore the range and diversity of experience or understanding amongst participants, rather than to estimate their frequency, statistical analyses were not performed on these data (33). This aim also informed our sampling approach regarding responses to one of the survey questions (“Can you describe why you wanted to have a dog?”) that generated a large volume of data. Through engagement with the data, the researchers considered code and meaning saturation (34). Once 3,000 responses had been coded, it was determined that new data rarely elicited new codes or generated different understandings. From this point, a quasi-random sampling approach was applied, whereby every 25^th^ response of the remaining data was coded. Overall, 39.9% (*n* = 3,215/8,050) of current owners' responses to this question were coded.

Regarding interview material, this article draws on our analysis of data where reasoning behind acquisition is discussed. We do not use the interview data in full because interviews incorporated additional topics not relevant to the current study (see interview guide in Supplementary Material).

For the presentation of qualitative findings in this article, direct illustrative quotes are used to give voice to the participants. To protect participant confidentiality, names have been omitted where mentioned, and unique identifier codes are used for each participant.

## Results

### Survey Results

In total, the survey was started 15,350 times. Following data cleaning and de-duplication, 11,265 of these responses were deemed “complete,” adhering to the conditions required for this study (respondents were required to be UK-based owners or potential owners) and suitable for data analysis. As this study aimed to investigate acquisition practices, we decided to exclude data from respondents who reported that they had bred their own dog(s) (*n* = 115) and those who stated that they were not involved in the decision to get their dog (*n* = 216). This left a total of 10,934 participants. Of these, 8,050 were current owners and 2,884 were potential owners. Of the 8,050 current owners, 27.4% (*n* = 2,205) were also potential owners (i.e., they answered “Yes” to a question in the survey that asked them to indicate if they were considering acquiring another dog “soon”).

#### Owner Demographics

The majority of survey respondents were female and the most common age category was 45–64 years (Table 1). There was a higher representation of people aged 45 years or older compared to those aged between 18 and 44 years (this split was 63:36 for current owners and 57:43 for potential owners). Most participants lived in England, but every country of the UK was represented.

**Table 1:** Demographic characteristics of survey respondents.

**Characteristic**	**Current owners**		**Potential owners**	
	**(*****n*** **= 8,050)**		**(*****n*** **= 2,884)**	
	***n***	**%**	***n***	**%**
**Respondent gender**				
Female	7,105	88.3	2,304	79.9
Male	865	10.8	551	19.1
Non-binary	8	0.1	1	0.0
Prefer to self-identify	8	0.1	3	0.1
Prefer not to say	64	0.8	25	0.9
**Respondent age group**				
18–24 years	474	5.9	216	7.5
25–34 years	1,248	15.5	562	19.5
35–44 years	1,206	15.0	453	15.7
45–54 years	1,916	23.8	573	19.9
55–64 years	1,821	22.6	599	20.8
65–74 years	1,126	14.0	374	13.0
75–84 years	189	2.4	83	2.9
85 years or older	7	0.1	5	0.2
Prefer not to say	63	0.8	19	0.7
**Respondent country of residence**				
England	5,549	84.2	1,991	82.4
Scotland	601	9.1	265	11.0
Wales	327	5.0	124	5.1
Northern Ireland	111	1.7	37	1.5
Unknown	1,462	22.2	467	19.3

#### Dog Demographics

Many current owners (62.8%) had acquired their dog within 5 years prior to survey completion (i.e., between 2015 and 2019). The remaining current owners had acquired their dogs between 2000 and 2014. At the time of acquisition, current owner's dogs ranged in age from 0 months to 19 years. Of the current owners, more than half (54.4%) had acquired their dog as a puppy (<=6 months), whilst a third acquired their dog between 1 and 6 years of age (32.5%). A further 6.6% had acquired their dog at seven years or older.

### Survey Results: Reasons, Factors and Influences Associated With Acquistion

**Figure 2:**
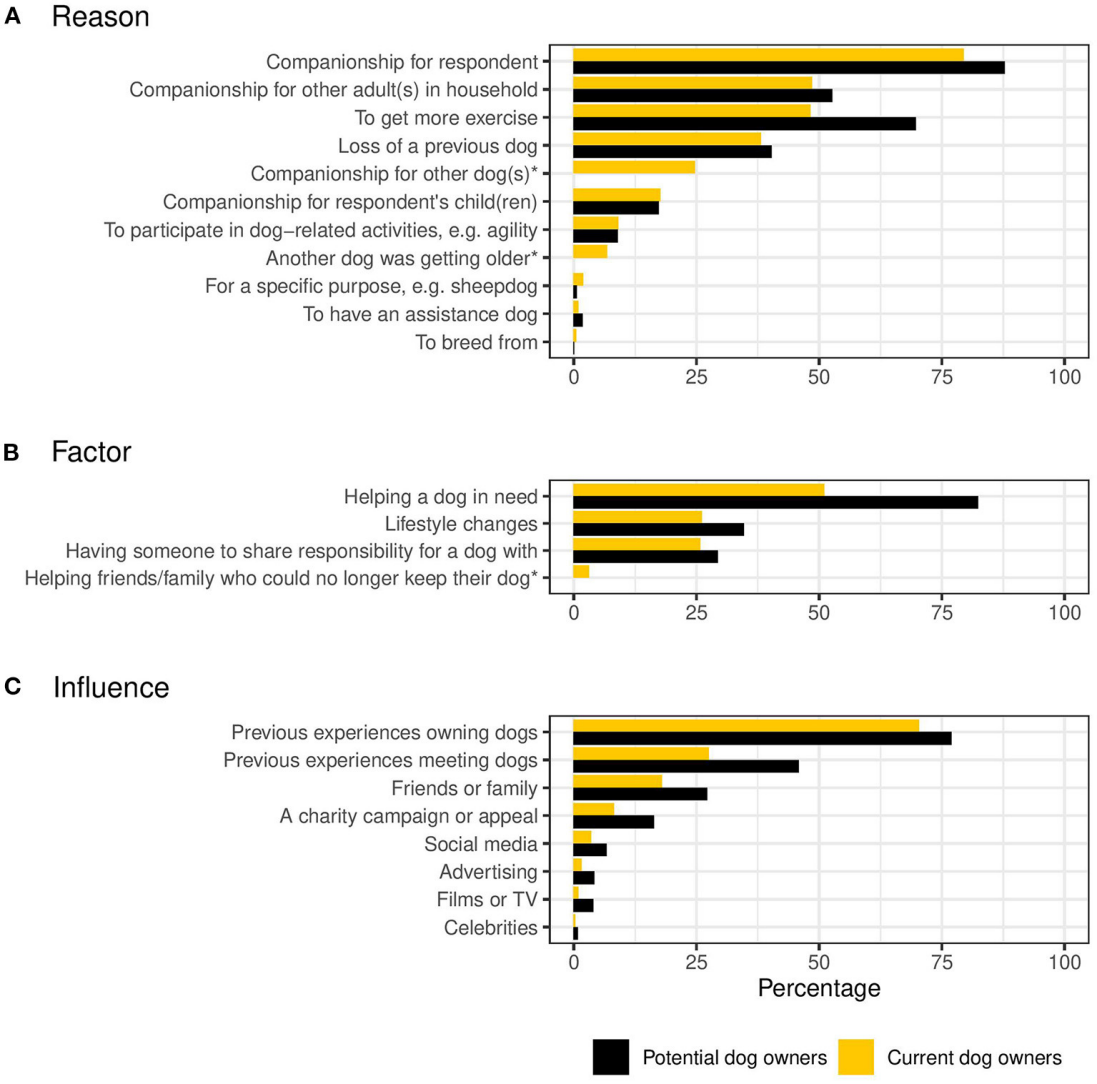
Reasons, factors and influences reported by the current owner group and the potential owner group. The asterisks (*) indicate response options that were only displayed to the current owner group. Motivational factors associated with dog acquisition as reported by the current owner group and the potential owner group. **(A)** Reasons for acquiring a dog. **(B)** Factors associated with the decision to acquire a dog. **(C)** Influences associated with the decision to acquire a dog.

Participants were asked to report which (if any) of the listed reasons for getting their dog were relevant to their desire to get a dog (either the last dog they had acquired, or a future dog, dependent on whether the respondent was a current or potential owner) (Table 2 and Figure 2A). Many respondents (53.5% of current owners and 63.0% of potential owners) selected either two or three reasons. A further fifth of respondents said that four of the listed reasons were involved in their decision to get a dog. The most common reason for wanting to get a dog, across both current and potential owners, was “companionship for respondent” (79.4 and 87.6%, respectively). For current owners, this was followed by “companionship for other adult(s) in the household.” Getting more exercise was also a reported reason for almost half (48.2%) of current owners. Amongst potential owners, the top four most frequently cited reasons for wanting to get a dog comprised the same assortment of reasons. However, the four most frequent responses were all reported by higher proportions of potential owners. Chi-squared analyses revealed significant differences between current and potential owners in their likelihood of reporting reasons for getting a dog. Potential owners were significantly more likely to report “companionship for respondent,” “companionship for other adult(s) in household,” “to get more exercise,” “loss of a previous dog,” and “to have an assistance dog” as reasons for wanting to get a dog than current owners. Current owners were significantly more likely to report “for a specific purpose (e.g., sheepdog),” and “to breed from” as reasons for getting a dog than potential owners.

**Table 2:** Reasons for wanting to get a dog.

**Reason**	**Current owners (*****n*** **= 8,050)**		**Potential owners (*****n*** **= 2,884)**		**Statistics**	
	***n***	**%**	***n***	**%**	**X^2^**	***p*-Value**
Companionship for respondent	6,395	79.4	2,531	87.8	97.46	<0.001
Companionship for other adult(s) in household	3,905	48.5	1,520	52.7	14.78	<0.001
To get more exercise	3,880	48.2	2,009	69.7	392.67	<0.001
Loss of a previous dog	3,065	38.1	1,161	40.3	4.17	0.041
Companionship for other dog(s)^*^	1,988	24.7	NA	NA	NA	NA
Companionship for respondent's child(ren)	1,421	17.7	499	17.3	0.16	0.692
To participate in dog-related activities, e.g., agility	734	9.1	258	9.0	0.06	0.811
Another dog was getting older^*^	550	6.8	NA	NA	NA	NA
For a specific purpose, e.g., sheepdog	154	1.9	18	0.6	21.957	<0.001
To have an assistance dog	79	1.0	53	1.8	12.35	<0.001
To breed from	36	0.5	3	0.1	6.10	0.013
None of the above reasons	321	4.0	29	1.0	NA	NA

* *These response options were not offered to potential owners, as they were anticipated to be only relevant for respondents who owned a dog at the time of survey completion and were thus reporting on actual acquisitions (i.e., “current owners”)*.

*Respondents could select multiple responses*.

In a separate question, participants were asked to report which (if any) of the listed factors were important considerations when deciding to get their dog (Table 3 and Figure 2B). Across both current and potential owners, the most frequently selected factor was “helping a dog in need,” reported by 51.1% of current owners and 82.3% of potential owners. “Lifestyle changes” and “having someone to share responsibility for a dog with” were each cited as important by one quarter (26.1 and 25.7%, respectively) of current owners. Slightly higher proportions of potential owners (34.6 and 29.3%) selected these factors, respectively. A quarter (25.4%) of current owners reported that none of the listed factors were important to them. Chi-squared analyses revealed significant differences between current and potential owners in their likelihood of reporting important factors involved in their decision to get a dog. Potential owners were significantly more likely to report “helping a dog in need,” “lifestyle changes,” and “having someone to share responsibility for a dog with” than current owners.

**Table 3:** Factors when deciding to get a dog.

**Factor**	**Current owners (*****n*** **= 8,050)**		**Potential owners (*****n*** **= 2,884)**		**Statistics**	
	***n***	**%**	***n***	**%**	**X^2^**	***p*-Value**
Helping a dog in need	4,110	51.1	2,374	82.3	858.42	<0.001
Lifestyle changes	2,101	26.1	999	34.6	75.81	<0.001
Having someone to share responsibility for a dog with	2,072	25.7	845	29.3	13.58	<0.001
Helping friends/family who could no longer keep their dog^*^	250	3.1	NA	NA	NA	NA
None of the above factors	2,046	25.4	243	8.4	NA	NA

* *This response option was not offered to potential owners, as it was anticipated to be only relevant for respondents who owned a dog at the time of survey completion and were thus reporting on actual acquisitions (i.e., “current owners”)*.

*Respondents could select multiple responses*.

Participants were also asked to report which (if any) of the listed influential factors were important in their decision to get their dog (Table 4 and Figure 2C). “Previous experiences of owning dogs” was the most frequently selected factor for both current and potential owners, cited by 70.3 and 77.0% of owners, respectively. The next most common response was “previous experiences of meeting dogs,” which was reported as important by almost half (45.6%) of potential owners and around one quarter (27.5%) of current owners. “Friends and family” were an important influence for 17.9 and 27.2% of current and potential owners, respectively. Chi-squared analyses revealed significant differences between current and potential owners in their likelihood of reporting influences involved in their decision to get a dog. Potential owners were significantly more likely to report each of the response options than current owners.

**Table 4:** Influences when deciding to get a dog.

**Influence**	**Current owners (*****n*** **= 8,050)**		**Potential owners (*****n*** **= 2,884)**		**Statistics**	
	***n***	**%**	***n***	**%**	**X^2^**	***p*-Value**
Previous experiences of owning dogs	5,660	70.3	2,220	77.0	46.54	<0.001
Previous experiences of meeting dogs	2,216	27.5	1,322	45.8	324.44	<0.001
Friends or family	1,442	17.9	785	27.2	112.80	<0.001
A charity campaign or appeal	660	8.2	470	16.3	149.39	<0.001
Social media	283	3.5	194	6.7	51.71	<0.001
Advertising	129	1.6	122	4.2	64.20	<0.001
Films or TV	74	0.9	116	4.0	117.92	<0.001
Celebrities	26	0.3	25	0.9	12.38	<0.001
None of the above influences	1,210	15.0	214	7.4	NA	NA

*Respondents could select multiple responses*.

### Interview Findings: Qualitative Themes Related to Motivations for Acquisition

A reflexive thematic analysis of interview transcripts and survey free-text responses identified fourteen themes. We abstracted these themes into three more implicit over-arching themes that represent key dimensions of reasoning behind dog acquisition: *Self-Related Motivation, Social-Based Motivation*, and *Dog-Related Positive Affect-Based Motivation*. These overarching themes were inspired by themes identified in previous research on a similar topic (16). Participants often reported multiple motivations for getting a dog, with some citing a combination of reasons across the overarching themes. Each overarching theme contained associated themes and sub-themes. These are presented alongside illustrative quotations in Supplementary Table 1 and discussed with accompanying narrative below.

#### Self-Related Motivation

The first overarching theme, *Self-Related Motivation*, comprised a collection of themes directly related to owner's lives and their expectations about dog's roles within their lives. Most of the themes contained within *Self-Related Motivation* highlight the multiple ways that participants perceive dogs–or aspects of dog ownership–to benefit the owner. A few additional themes within *Self-Related Motivation* (*Owner's Ability to Care for a Dog* and *The Right Time*) relate to practical concerns about the owner's capacity to look after a dog, and factors that affect the timing of acquisition, respectively. Finally, *Owner's History with Dogs* was a further theme within *Self-Related Motivation* which was about the influence of previous experiences with dogs in driving the desire to get a dog.

##### Valued Aspects of Human-Dog Relationships

Many participants referred to prized elements of the relationships and interactions between humans and dogs. The companionship provided by a dog was a commonly reported reason for getting a dog. Some participants described companionship in general terms, such as having someone to accompany them through life. Others referred to their desire for a dog's company during specific activities, commonly whilst on walks, or in specific places, such as in the home.

Positive relationships with dogs were widely anticipated, with some describing their desire to establish bonds, experience mutual love and friendship with their dog. The following response illustrates this: “*I love the bond u [sic] build*.” (Survey–Potential owner−3494).

For some participants, dog ownership was sought for the opportunity to love and provide care for another being. As one participant responded: “*Having a dog is having someone to love and look after*.” (Survey–Potential owner−8210). This caring aspect of the relationship was a source of pleasure for some participants, as illustrated in the following response in which a participant compared looking after their dog to looking after their children: “*I like the grooming aspect. And feeding them. And caring for them. (…) I get more pleasure out of looking after my dogs than my children! [laughs]*.” (Interview–Current owner – A1KH08C609).

##### Benefits to Human Health and Wellbeing

Dogs, or aspects of dog ownership, were widely perceived to promote human health and wellbeing, with anticipated benefits on both mental and physical health. Regarding mental health or wellbeing, many participants reported that dogs were desirable for their capacity to improve mood, provide a sense of purpose, or mitigate feelings of loneliness. These benefits were often described as being achieved through the dog's company, the emotional support dogs offer, and the owner's caregiving responsibilities and routine that were understood as a feature of ownership. As an example of the latter point, one participant associated the routine of walking their dog with positive impacts on mental health: “…*I also think dogs are fantastic for our own mental health, helping us get out of the house regularly for fresh air/exercise*.” (Survey–Potential owner−1108). A dog's positive impact on mental health was sometimes associated with the management of a specific health condition or illness. Anxiety and depression were commonly reported mental health conditions that participants associated with their desire to get a dog. As an example of the perceived, or anticipated, benefits of dog ownership related to anxiety and depression, one participant noted: “*I have depression and anxiety and have found walking my family's dogs and spending time with them helps me. I feel having a dog would give me purpose and routine and would also help me to leave the house and get exercise*.” (Survey–Potential owner−3735).

In addition to mental health benefits, participants linked dog walking with other anticipated health and wellbeing benefits. Many participants suggested that they sought a dog to increase their exercise and improve their physical health. As well as facilitating exercise, dog walking was reported to foster human–human social connection, with dogs acting as social conduits. For example, one participant reported: “*When you own a dog you become known as that dog's mum or dad, when you are out on walks other dog owners etc always see the dog first then you. When you loose [sic] your dog it's like you disappear as well*.” (Survey–Current owner−1817). The social aspect of walking, that included meeting and talking to other dog owners, was widely desired and its value was emphasized by one participant in the following example: “*I work from home so she is a companion for when I'm at home and she gets me out and about meeting people when we go out for walks. Without her my life would be isolated*.” (Survey–Current owner−3472).

##### Dogs Enrich Owner's Life

In addition to health- and relationship-related benefits, participants expressed a more general notion that dog ownership enhances the quality of owner's everyday lives. Dogs were often reported to bring about, or increase, fun or enjoyment in an owner's life. Sometimes this was associated with activities, such as dog walking. For example, one participant reported: “*Myself and my partner love long walks in the countryside and having a dog makes it that much more enjoyable*.” (Survey–Current owner−9943). Some participants suggested that living with dogs not only enriches their lives but “completes” them. For example, one participant commented: “*When you have owned dogs previously that have died ones (sic) life is like an incomplete jigsaw*.” (Survey–Potential owner−59). However, participants expressed variation in the degree of importance they considered dog ownership to have on their lives. For instance, some believed that, while dogs improve their lives, they are not integral to their existence, as the following response illustrates: “*My dogs are not my life, but that [sic] make my life better*.” (Survey–Current owner−1029).

##### Dogs Mediate Owner's Self-Identity

Some participants emphasized the role that dogs play in constructing and affirming their sense of self. Dogs helped some respondents to understand and validate how they saw themselves, often as a person very fond of dogs (or animals more generally). In some cases, participants identified themselves as “dog-” or “animal-people.” Beyond feeling an affinity with dogs or animals, some participants stated that a dog is essential to their sense of self. This is illustrated in the following response: “*I can't live without them, they are part of who I am*.” (Survey–Current owner−2458). A further way in which dogs were understood to contribute to an owner's sense of self-identity was more explicitly focused on how the participant felt they were viewed by other people. These participants shared a perception that dogs mediated how other people viewed them, particularly in public spaces. For example, a few participants reported that being accompanied by a dog provided them with a legitimate reason for walking alone, as illustrated in the following quote: “…*I need to get out and walk. I can't do it without a dog. It's just impossible. You can't go for a walk. I mean I look at people weirdly if they're walking in the park and they don't have a dog with them. I'm always a bit suspicious. Especially if a man's walking alone on his own. I'm like, ‘what's wrong with you? Why don't you have a dog? Where's your dog?'*” (Interview–Current owner–B1RM0403).

##### Desire to Participate in Lifestyle Associated With Dog Ownership

The desire for a dog was often associated with lifestyle aspirations. This often included references to participants' desires to walk with a dog: “*I wanted a dog to go on walks with*.” (Survey–Current owner−609). Some participants were already keen walkers and sought a dog to join them for this activity, suggesting that this made walking more pleasurable, or helped them to explore new places. For some others, having a dog was anticipated to increase their walking frequency, as the following response illustrates: “*Having a dog will encourage me to get out more and get back to walking*.” (Survey–Potential owner−6981). Some participants also expressed interest in the opportunity to take part in dog-related activities, such as sports (e.g., agility) and training. The idea of training appealed to some owners, for example in the following response: “*I'm quite a project person, so the thought of training a dog is really exciting to me. I'm really into that…we're a bit outdoorsy people as well, we love walking*.” (Interview–Potential owner–B2RM0301).

##### Dogs as Family Members

Many participants expressed reasons for getting a dog that were associated with the roles they perceived dogs to play within the family unit. Dogs were widely described as “part of the family,” with some participants noting that a dog “completes” the family. For example, one participant reported: “…*me and my wife now already have children so we decided to have a dog instead of having a baby. This completed our family*.” (Survey–Current owner−2219). Especially for participants who did not have children in the household–whether through choice or not–dogs were often desired for the opportunity they offer the owner to nurture another being: “*Someone to care for when I found out I couldn't have children*.” (Survey–Current owner−918).

Participants expressed diverse views regarding the extent to which they considered dogs and children to be synonymous. Some described dogs as alternatives, or akin, to children, as illustrated in the following example: “*A dog can be a substitute child when your own children and [sic] fled the nest*” (Survey–Current owner−7357). Conversely, others were explicit that dogs were not synonymous with children. For instance, one participant noted: “…*we wanted to extend our family without having a child*.” (Survey–Current owner−4440). However, dogs were still widely considered family members even if distinguished from human children, as demonstrated in the previous and following quotes: “*We don't have any children and that's a choice for us. So instead, they're not our third babies, because they're animals, I should say that readily. But we have an animal family*.” (Interview–Current owner–B1RM0601).

##### Functional Roles Performed by Dogs

Occasionally dogs were acquired to perform a functional role, for example traditional working tasks (e.g., herding sheep). Other functional roles dogs were sought for included providing protection or security to the owner and/or home and service roles (e.g., as therapy or assistance dogs). Some respondents did not desire a formal working dog as such (i.e., a dog trained to perform a specific task), but referred to dog's behavioral or physical characteristics that they associated with the performance of functional roles, commonly protection. This is illustrated in the following response: “*They're great companions and usually protective too*.” (Survey–Current owner−5109).

##### Owner's Ability to Care for a Dog

In addition to self-related reasons for wanting to get a dog, the overarching theme *Self-Related Motivation* also provided insights into the self-related factors that motivate people to follow through with this intention. Participants' assessments of whether they were able to sufficiently care for a dog were frequently cited, with the impact of various personal lifestyle or circumstantial factors related to a person's (or household's) ability to care for a dog considered. The ability to care, or provide a “good” home, for a dog was considered practically (e.g., available time to dedicate to a dog, considering work commitments and lifestyle; suitability of living accommodation), financially, and emotionally (e.g., after grieving the loss of a previous dog). The following response incorporated several of these factors: “*Our last dog dies [sic] 7 years ago and it's only now that we feel able to have another dog. We are retired but very active and feel we could offer a rescue dog a lovely home where it would be loved unconditionally*.” (Survey–Potential owner−9267). Given participants' consideration of the dog's needs and perspective, in their assessment of their ability to care for a dog, the theme *Owner's Ability to Care for a Dog* thus sometimes also fitted into the overarching *Pro-Social: Dog Related* theme.

Time, or lack of it, was a frequently reported factor associated with participant's assessments of their ability to care for a dog, and their subsequent decision about whether to get a dog. Owners' working hours, routine, or location (e.g., working from home) were often linked to the time an owner had available. The available time an owner had was sometimes associated with their ability to be present at home, to ensure a dog was not left home alone for long periods. An example of this is provided in the following response: “*I work a few hours a day and get school holidays off, so the dog wouldn't be alone too long*.” (Survey–Current owner−3616). Sometimes participants cited their support network (e.g., friends and family) as helping to care for a dog and thus making ownership possible.

##### The Right Time

The desire to get a dog had been a long-held wish for many participants. Sometimes this had been an aspiration since childhood, as in the following example: “*I'd always wanted one from a kid*.” (Interview–Current owner–B1RM1201). Acting on the intention to get a dog was often motivated by circumstantial factors that produced a belief that it was the “right time” to get a dog. The “right time” was often understood to be the product of an owner's circumstantial factors that meant they had the capacity to care for a dog. An example of this is provided by one participant who described how a shift in their work arrangements created a “good time” to get a dog: “*At the moment I'm on what's known as a sabbatical, study leave, which means that I can work lots from home. So I'm not having to go to the office. And I just thought it would be a good time, get all the ducks in a row so that the dog's got someone to play with, you know, someone to look after her and everything*.” (Interview–Current owner–B1KH1101). Life transitions associated with changes in lifestyle (e.g., retirement or relocation) and the loss, or aging, of other pets (typically, but not exclusively, dogs), were widely cited as catalysts for dog acquisition.

##### Owner's History With Dogs

Many participants cited their previous experiences with dogs as influencing their desire to get a dog. Growing up with dogs was widely reported to motivate this yearning, as in the following example: “*I have always wanted a dog as I grew up around them*.” (Survey–Current owner−526). For people who had lived with dogs for much, or all, of their life, being in a home without a dog was unsettling. For example, one participant explained: “*When you've had dogs for 20 years it's, yeah, it's quite difficult coming in the house not having a dog here*.” (Interview–Potential owner –B1KH1102). Some others were motivated by their experiences with dogs through those owned by friends and family, or through time spent working with dogs, either in a professional or voluntary capacity.

In some cases, participants reported having a prior relationship with dog they acquired. For instance, where dogs had been previously owned by someone in the participant's social network. In addition, dogs that were initially fostered by participants were subsequently acquired on a permanent basis. This was typically motivated by an attachment that had formed between participant and dog.

#### Social-Based Motivation

This overarching theme encompassed themes about reasoning for getting a dog that was influenced by others (human or dog). Some social-based motivations were about a desire to benefit others. These motivations were categorized as either *Pro-Social: Dog-Related* or *Pro-Social: Human-Related*, depending on whether the acquisition was anticipated to benefit a dog or other human (beyond the owner). An additional theme, *Influenced by Social Network*, was characterized by decision-making that involved others, but was not reported to directly benefit them.

The name of this overarching theme, *Social-Based Motivation*, differs slightly from that of a similar theme (”Prosocial-Based Motivation”) identified in a previous study (16) that our overarching themes were inspired by. This change was made to better reflect the scope of the themes contained within our overarching theme *Social-Based Motivation*, acknowledging that some of the content within this overarching theme does not relate to reasoning that intends to benefit others.

##### Pro-social: Dog-Related

A primary pro-social motivation was the desire to help a dog, typically one considered to be “in need.” Some participants described helping a dog by acquiring them as a moral act, as illustrated in this participant's response: “*I've always rescued animals and wanted to save a life*.” (Survey–Current owner−150). Participants' decisions about whether to get a dog were sometimes linked to their perception of dog happiness and wellbeing. This included ideas about what was “best,” “fair” or “right” for a dog. For many participants who already had a dog/s in their household, another dog was sought as a companion, friend, or playmate for their current dog/s, often with the expectation that this would benefit their current dog's wellbeing. For example, one participant reported: “*We had lost another dog, and our surviving dog was extremely lonely and needed a new companion*.” (Survey–Current owner−2502). In some cases, a new dog was sought to provide emotional or behavioral support for a current dog, for instance boosting their confidence: “*We already had a very timid rescue dog who needed a more confident dog to help him*.” (Survey–Current owner−3546).

##### Pro-social: Human-Related

For some participants, dogs were sought for the benefit of a person beyond the primary owner–most often children. Sometimes a child had expressed a desire for a dog, as in the following response: “*My daughter has wanted a dog since she was 3 yrs old and we have always put her off, now seems the right time and we have put a lot of thought into it*.” (Survey–Potential owner−9370). Dogs were felt to have a positive general influence on children growing up. Sometimes this was associated with the participant's own experience of growing up with a dog, as this participant commented: “*I always had a dog growing up and wanted my daughter to discover how enjoyable it is.”* (Survey–Current owner−169). Some participants believed that dogs help support children's physical, health, emotional, and social needs and development. The perceived capacity of dogs to teach children about responsibility was emphasized. For example, one participant said: “*Great for kids to grow up with dogs to teach responsibility*.” (Survey–Potential owner−2901). In addition to benefitting children specifically, participants reported that dogs were sought to bring the family together around a shared interest and increase quality time spent together, often through the routine activity of walking: “*I think it will add to our family bringing a love focus. It will make me get out and walk more during the week and provide family focus for weekend walks*.” (Survey–Potential owner−9019).

In addition to benefitting family members within the household, some participants stated that they wanted to get a dog for the sake of other people outside the household who were unable to take on the full responsibilities of ownership themselves. For example, one participant commented: “*My grand daughter was desperate for a dog but could not have one at her own home.”* (Survey–Current owner−5452). As well as grandchildren, some participants referred to acquiring a dog to benefit older parents who could no longer care for one of their own.

##### Influenced by Social Network

Deciding to get a dog was often described as a joint, or family, decision. Some participants reported that the decision get a dog was initiated by another household member (i.e., a partner or child). Some respondents commented that the individuals involved in shared decision-making had differing levels of interest in acquiring a dog. For example, one participant commented: “*My husband keeps saying ‘Oh we don't need another dog.” But I would like another dog*.” (Interview–Potential owner–B1KH1104).

Some participants explained that they were inspired or encouraged to get a dog by someone else beyond the immediate household. Friends and family were commonly mentioned influencers. In some cases, friends and family suggested the respondent get a dog when they expressed concern about the respondent's mental health. For example, one participant explained: “*I had suddenly lost my partner in an accident and then my mum 14 months later in another accident. My nieces wanted me to have a reason to go out other than to work*.” (Survey–Current owner−4300).

#### Dog-Related Positive Affect-Based Motivation

This overarching theme was characterized by a cluster of sub-themes about positive feelings toward dogs that many participants expressed. As well as broad references to dogs in general, positive sentiments were sometimes expressed toward animals more broadly, or in favor of a particular dog breed more specifically. Various dog-related qualities participants valued were mentioned, which commonly included their “loving” and “loyal” nature. As an example of this, one participant noted: “…*they're loving loyal companions who become one of the family*.” (Survey–Potential owner−9170).

Although some participants reported a fondness toward animals more generally, our findings offer some insight into why dogs might be favored over other species, as participants distinguished the roles and qualities of dogs from those of cats. For example, one participant reported: “*When the second of my dogs died, my life was simply incomplete. My lovely rescue cat [name omitted], filled some of the void but cats have a very different role than a dog*.” (Survey–Current owner−3120). The quality of interactions with–and support derived from–dogs was often perceived as unique, from other human–animal relationships, with dogs perceived as providing a distinct kind of companionship. This is illustrated by the following response: “*I think their, like, companionship, is just like no other pet. I could get a cat. But they're not as loving. I don't know. Well the cats I've had aren't anyway*.” (Interview–Current owner–B1RM0402).

## Discussion

The findings from this study indicate that people are motivated to acquire dogs for a variety of self-related or social-based reasons, in addition to holding positive feelings and attitudes toward dogs. The diversity of reasons for acquisition reported in this study, and the nuances in participants' perceptions of the roles that dogs play within their lives, suggests that the flexibility of dogs to be and mean different things to different people helps to explain their broad appeal.

Whilst a variety of reasons for acquisition were reported in this study, the majority of survey respondents sought dogs to provide companionship to the owner and/or wider household, with few dogs acquired to perform specific working roles, such as providing security or assistance. The importance of companionship as a reason for acquisition was confirmed by both the quantitative and qualitative findings from this study. This supports previous evidence highlighting companionship as a key reason for dog acquisition (11–14). However, the current study extends existing understanding of the primary motivating role companionship plays in the purchasing of puppies (11), by confirming its importance in the acquisition of older dogs too.

The dog's ability to provide companionship to their owners is a result of the close bonds they form with people (35). This mechanism is indicated in the current study's qualitative findings, as many respondents described their relationships with dogs (current or anticipated) in emotive terms such as “love” and “friendship.” As well as the perceived benefits of dog ownership for the human, a wish to confer benefits on a dog, for instance through providing them with care and affection, was also cited in qualitative responses. This finding supports the “opportunities for nurturance” aspect of the non-human social support theory, which is an adaptive argument hypothesized to explain the development of pet keeping (10, 36).

Another element of the social support theory is “emotional support,” or the feeling that one is cared for by others or can rely on others for comfort (36). Our qualitative findings indicate that the emotional support anticipated to be gained through dog ownership was an important factor driving dog acquisition amongst our participants. As well as mental health, this study's results suggest that owners anticipate physical health benefits to accompany dog ownership: respondents frequently cited the desire “to get more exercise” as a reason for wanting to get a dog. These findings indicate a perceived “pet effect” amongst respondents: the idea that pet ownership will promote human health and happiness. Previous studies have reported human health benefits associated with dog interaction or ownership, such as reductions in stress, anxiety, and loneliness, and increased physical activity (37–40). However, reviews of research on the effect of pet ownership on human loneliness, depression, and obesity have produced mixed results, with most research not demonstrating evidence linking pets and human health (41–43). In some cases, the responsibilities associated with certain pets may instead lead to a caregiving burden (44). Nevertheless, the health benefits of dog ownership are promoted to potential dog owners in media reports, recently exemplified during the COVID-19 pandemic (45). It is possible that owners' expectations or beliefs around the social or emotional support dogs may provide are linked to media coverage about the positive impact of pets on people, with fewer stories reporting on the downsides of living with pets (46). Understanding how a potential owner's expectations about the role dogs have in/on human health match with the reality of dog ownership, and/or affect future satisfaction with the relationship, is an important future research area. It is likely that a dog's impact on human health and wellbeing varies between dog–owner dyads, according to individual needs and circumstances (47), as well as changing over time within individual dyads, affected by changing circumstances such as a dog's aging (48). Furthermore, given the emphasis on dogs' anticipated role in providing emotional support indicated in this study's qualitative findings, future research could consider potential welfare implications for dogs arising from the demands made upon them by owners who acquire them to provide emotional support (49).

Findings from this study suggest an understanding of dogs as key family members. Their perceived significance in this role is indicated by remarks that the family would not be complete without a/their dog. This finding is consistent with research indicating that owners consider pet dogs as part of the family (18, 50–54). Previous literature suggests that, for many people, it is the dog's behavioral flexibility that makes them such appealing family members that can even supersede children: ‘they are more easily mobilized [than children], require less investment, and to some degree can be shaped into whatever you want them to be–a best friend, a lover, an occasional companion' ((55), p.302). In line with this, some participants in the current study expressed a preference to share their lives with dogs over children. However, this study identified diversity in understandings of the role of dogs in the family vis-à-vis children. Further research to explore the nuances in how owners understand their dogs' roles within the family is warranted.

The qualitative data analysis performed in this study identified a further reason that motivates some people to acquire dogs, concerning the roles dogs play in people's understanding and projection of themselves. These insights suggest that dog ownership is desirable because it has the capacity to influence the way others perceive the owner. For example, respondents described being alone in public spaces, such as parks, and feeling concerned they may arouse suspicion from other people. Respondents suggested that being accompanied by a dog can help establish themselves as having legitimacy in such spaces. This motivation can be understood through the theory of “impression management”: a process through which individuals work to create an image of themselves to influence the perceptions of other people about them (56).

In addition to identifying a variety of perceived advantages associated with dog ownership that drive owners to acquire dogs, this study's results highlight additional factors that influence the decision to act on the desire to get a dog. Lifestyle changes were a cited factor influencing the decision to acquire a dog for more than one quarter of owners (both current and potential) in this study. Our qualitative findings offer deeper insights on this topic, revealing a variety of lifestyle changes and events reported to influence respondent's decisions to acquire a dog. Common examples included relocation and employment-related changes. Such factors were often reported to provide the owner with the resources (e.g., time, space, money, support, or proximity to walking locations) that they felt positioned them as able to care for a dog. These findings are supported by a recent study of people who purchased puppies during the COVID-19 pandemic, in which many respondents (86.7%) reported being influenced to do so by having more time to care for a dog at this (11).

Previous research suggests that people have an innate instinct to care for dogs (57, 58). This may be a mechanism that helps to explain why many owners in the current study reported that their decision or intention to acquire a dog was motivated by a desire to help a dog in need. In the current study, potential owners were significantly more likely to report being motivated by a desire to help a dog in need. However, this finding requires cautious interpretation: due to this study's promotion via the channels of the UK's largest dog welfare charity, the sample may be biased toward owners with certain beliefs or interests, for example in helping vulnerable dogs. Existing literature suggests that ethical motivations influence people who choose to acquire a rescue dog (59). However, given the sample limitations noted, future studies are needed to investigate the role of different motivations, such as the desire to help a dog in need, in influencing distinct types of owners, such as those who adopt dogs vs. those who buy puppies.

In the current study, previous experience with dogs (either through previous ownership or dogs that respondents had met) was the most commonly cited influence affecting decisions to get a dog. This is consistent with findings from previous studies conducted in the Netherlands and the USA, in which previous dog experience was a major reported influenced in respondent's decisions to acquire their dog (14, 60). The current study also identified friends or family as an important reported influence in the decision to get a dog. This is in line with findings from previous research that highlight the influential role friends or family may play in acquisition decisions, for instance as a common source of pre-acquisition information, as well as influencing decisions around breed choice (11, 61). Understanding the nuances of discussions between potential dog owners and their family and friends would be a worthwhile aim of future research which may enable the development of more targeted interventions to promote responsible decision-making around dog acquisition. This study's insights are in line with evidence that has indicated the major role social norms (e.g., around what is considered “fashionable”) play in influencing human behavior, including potential dog owner's decision-making more specifically (62). However, in the current study, media (social media, advertising, films and TV, and celebrities) were infrequently reported as influences, amongst both current and potential owners. Whilst this finding is consistent with a previous study in which celebrity ownership was not reported as influential in owner's decisions to acquire their chosen breed (63), it is contrary to other research that identified a correlation between film releases and the popularity of featured dog breeds (64). If media and celebrity culture do influence dog acquisition decision-making, it is likely that this operates at a subconscious level, with random drift proposed as a potential hypothesis to explain pet keeping behavior (65). This may explain why, despite answering sincerely, few respondents report it as an influence in self-report survey studies. Alternatively, respondents may be reluctant to admit that media could influence their decision-making. The contradictory findings on this topic suggest that future studies looking to understand the role of media influence on potential owners' decision-making would benefit from an approach that goes beyond only self-report surveys. Furthermore, as younger people are heavier users of social media (66), and because this study had a greater representation of people aged 45 years or older compared to those younger than 44 years, future research would benefit from investigating whether age-related differences influence the impact of social media on prospective dog owners.

This study's findings also demonstrate that current ownership status influences motivations for dog acquisition, and likely also expectations of ownership. People who did not own a dog at the time of survey completion (i.e., potential owners) were significantly more likely to report “companionship for themselves,” “companionship for other adults in the household,” and “to get more exercise” as reasons for wanting a dog than current owners reporting retrospectively. This finding might suggest that motivations for getting a dog change throughout the process of acquisition and/or ownership. Further longitudinal study of the acquisition process would be valuable to investigate this and could enhance our understanding of shifting desires and expectations of potential owners. This finding is also comparable with a study that compared expectations of dog ownership among potential owners with different dog ownership statuses (never owned a dog, previous owner, current owner) and found that only previous owners had greater odds of expecting to walk more than people with no previous ownership experience (15). However, as our study asked current owners about their most recent prior acquisition, rather than their expectations toward prospective acquisitions, as in Powell et al. (15), a direct comparison between the two is not possible. Nevertheless, both studies indicate that ownership status affects expectations of ownership. It is possible that the current owners in this study were influenced by some recall bias as they reported retrospectively about their most recent acquisition. Experiences of ownership since acquisition may have clouded their recollection of what motivated them *prior* to that acquisition. This study's findings might therefore indicate differences between the anticipated experience of dog ownership and the reality, possibly suggesting that potential owners are highly aspirational in their expectations of dog ownership and its benefits. Ensuring potential owners' expectations of ownership are realistic is important to optimize dog and human welfare. Pre-acquisition counseling provided by veterinarians offers an opportunity to limit unrealistic expectations through evidence-based discussions on the realities of dog ownership (59). Previous evidence suggests that although almost half of UK pet owners (45%) would be interested in a pre-acquisition consultation with a veterinarian, only 3% had already had one (67). To maximize potential impact, future interventions developed to achieve this aim could usefully consider the other information sources people commonly use prior to acquisition, which include websites, books, and friends and family (61).

## Strengths and Limitations

To the authors' knowledge, this is the largest study to investigate motivations for dog ownership amongst UK dog owners. Our mixed-methods approach generated qualitative data that built on the statistical analyses of the quantitative survey data. The integration of quantitative and qualitative data, where each confirmed the findings of the other, gave our findings greater credibility. In addition to a high quantity free-text approach for the analysis of survey-generated qualitative data, semi-structured interviews yielded deeper insights that expanded understanding about why so many people acquire a dog at some point in their lives. This study also benefitted from collecting data from both current and potential owners, enabling comparison of these populations which identified significant differences.

However, this study has several limitations. Firstly, the subgroup of our sample who were current owners, and thus reflecting on historical events, may have been at risk of recall bias. These owners were asked to recall an event (i.e., their most recent dog acquisition) that ranged in distance into the past (from <1 month to 19 years), depending on when they got their dog.

A further limitation of this study concerns the self-selection process of the survey sample. This sample consisted of UK dog owners (or potential owners) who had access to the Internet and were primarily female. This gender bias reflects other research in the field of human–animal studies (68–70), but future studies should attempt to recruit a more gender-diverse sample of respondents to minimize sampling bias. This study's sample may also bias toward owners with certain beliefs or interests, for example in dog welfare. Caution should therefore be observed if attempting to generalize to other populations.

Finally, this study did not examine the association between owner's motivations for dog acquisition and demographic characteristics (e.g., owner age, gender), so future research should explore if reasoning varies across demographic groups. This information could help target interventions around responsible acquisition to specific populations.

## Conclusion

In this study of UK dog owners, decisions to get a dog were driven by self-related and social-based motivations, in addition to positive feelings toward dogs. A majority of respondents reported that their decision to get a dog was influenced by a desire for companionship for the owner, helping a dog in need, and previous dog ownership. Participants often reported a combination of reasons, factors, and influences involved in their decision to get a dog, and this study's findings suggest that, in some cases, dogs may be sought to fulfill multiple roles in their owner's life.

This study's findings could be used to direct the development of interventions around responsible dog acquisition, to support potential owners to optimize both dog and human welfare. The presented insights might also help those working in the dog rescue environment to improve their appeal to potential owners. For instance, rescue organizations could adapt their messaging to emphasize how potential owners might help dogs in need by considering them as a source of dogs; potentially increasing adoption rates. Furthermore, this study's qualitative findings provide useful insights into reasons and factors driving dog acquisition to inform future large-scale surveys on dog acquisition.

This study has suggested various avenues for future research on the topic of dog acquisition. Future investigation is necessary to explore whether the experience of living with a dog is consistent with owner expectations around the roles of dogs in their life. Further work could also usefully explore whether different reasons for acquisition are associated with acquisition-related decisions (e.g., breed and source of dog), the subsequent treatment of dogs, and the quality of human–dog relationships.

## Data Availability Statement

The raw data supporting the conclusions of this article will be made available by the authors upon reasonable request.

## Ethics Statement

The studies involving human participants were reviewed and approved by Dogs Trust Ethical Review Board. The patients/participants provided their written informed consent to participate in this study.

## Author Contributions

KH, RM, and RCh collected and compiled the data, conducted the qualitative analyses, and interpreted the data. RM and RCh conducted the statistical analyses. KH drafted the manuscript. All authors conceived and designed the study and contributed to manuscript revision, read, and approved the final version.

## Funding

This work was funded by Dogs Trust and the authors are salaried employees of Dogs Trust. The authors would like to thank Dogs Trust for providing funding for Open Access publication.

## Conflict of Interest

The authors declare that the research was conducted in the absence of any commercial or financial relationships that could be construed as a potential conflict of interest.

## Publisher's Note

All claims expressed in this article are solely those of the authors and do not necessarily represent those of their affiliated organizations, or those of the publisher, the editors and the reviewers. Any product that may be evaluated in this article, or claim that may be made by its manufacturer, is not guaranteed or endorsed by the publisher.

## Footnotes

^1^We acknowledge the controversy surrounding terminology used to describe animals and the people who live with and care for them (3). For the sake of brevity and clarity, we use the terms “pet” and “owner” throughout this article, but our use of these conventional terms does not imply endorsement of domination.
