# Supplementary material for: Why Do People Want Dogs? A Mixed-Methods Study of Motivations for Dog Acquisition in the United Kingdom
Source: Front Vet Sci. 2022 May 10;9:877950. doi: 10.3389/fvets.2022.877950 (PMC9127952; doi:10.3389/fvets.2022.877950)
Supplement: Supplementary file 1 [file Data_Sheet_1.docx]

Supplementary Material

**“Choosing My Dog” survey questions relevant to Motivations for Acquisition study**

***Current and future dog ownership status***

**ALL respondents**

| **Question** | **Response options** |
| --- | --- |
| Do you currently own a dog? | - Yes: I currently own at least one dog - No: I don't own any dogs at the moment |
| Are you seriously considering buying or adopting a new dog soon? | \| - Yes: I'm actively looking for a new dog at the moment \| \| --- \| \| - Yes: I am seriously considering getting a new dog in the next six months but I'm not actively looking at the moment \| \| - Yes: I am seriously considering getting a new dog but this probably won't be in the next six months \| \| - No: I'm not currently considering getting a new dog \| |

***Demographic variables***

**ALL respondents**

| **Question** | **Response options** |
| --- | --- |
| What is your gender? | - Female - Male - Prefer not to say - Prefer to self-identify (please specify): |
| What age group do you fit into? | \| - 18 - 24 years \| \| --- \| \| - 25 - 34 years \| \| - 35 - 44 years \| \| - 45 - 54 years \| \| - 55 - 64 years \| \| - 65 - 74 years \| \| - 75 - 84 years \| \| - 85 years or older \| \| - Prefer not to say \| \|  \| |
| What is the first part of your postcode? Please leave blank if you would prefer not to say. | [Free text] |

***Motivations and influences for dog acquisition***

**CURRENT owners**

| **Question** | **Response options** |
| --- | --- |
| Can you describe why you wanted to have a dog? | [Free text] |
| What were your reasons for wanting to get a dog? Please select all that apply. | \| - Companionship for me \| \| --- \| \| - Companionship for other adult(s) in my household \| \| - Companionship/a pet for my children \| \| - Companionship for another dog(s) \| \| - Loss of a previous dog \| \| - As another dog was getting old \| \| - Getting more exercise for myself \| \| - Having an assistance dog for myself \| \| - Having a dog to breed from \| \| - Having a dog to do a specific purpose (e.g. gundog, sheepdog, etc.) \| \| - Participating in dog-related activities (e.g. agility, Canicross, etc.) \| \| - None of the above - Other reasons (please specify): [free text] \| |
| Were any of these factors important when deciding to get a dog? Please select all that apply. | \| - Lifestyle changes (e.g. moving house, changing job, etc.) \| \| --- \| \| - Having someone to share responsibility for a dog with \| \| - Helping a friend/family member out who could no longer keep their dog \| \| - Helping a dog in need (e.g. rescuing or rehoming) \| \| - None of the above - Other factors (please specify): [free text] \| |
| Were any of these important in influencing your decision to get a dog? Please select all that apply. | \| - A charity campaign or appeal \| \| --- \| \| - Advertising (e.g. brands using dogs in adverts) \| \| - Celebrities \| \| - Films or TV \| \| - Social media \| \| - Friends or family \| \| - My previous experiences of owning dogs \| \| - My previous experiences of meeting dogs (e.g. through friends or when out walking) \| \| - None of the above - Other influences (please specify): [free text] \| |

**Current owners who were also looking to acquire another dog**

| Can you describe why you want to have another dog? | [Free text] |
| --- | --- |

**POTENTIAL owners**

| Can you describe why you want to have a dog? | [Free text] |
| --- | --- |
| What are your reasons for wanting to get a dog? Please select all that apply.  Are any of these factors important when deciding to get a dog? Please select all that apply. | \| - Companionship for me \| \| --- \| \| - Companionship for other adult(s) in my household \| \| - Companionship/a pet for my children - Loss of a previous dog - Getting more exercise for myself - Having an assistance dog for myself - Having a dog to breed from - Having a dog to do a specific purpose (e.g. gundog, sheepdog, etc.) - Participating in dog-related activities (e.g. agility, Canicross, etc.) - None of the above - Other reasons (please specify): [free text] \|  \| - Lifestyle changes (e.g. moving house, changing job, etc.) \| \| --- \| \| - Having someone to share responsibility for a dog with \| \| - Helping a dog in need (e.g. rescuing or rehoming) \| \| - None of the above - Other factors (please specify): [free text] \| |
| Have any of these influenced your decision to get a dog? Please select all that apply. | \| - A charity campaign or appeal \| \| --- \| \| - Advertising (e.g. brands using dogs in adverts) \| \| - Celebrities \| \| - Films or TV \| \| - Social media \| \| - Friends or family \| \| - My previous experiences of owning dogs \| \| - My previous experiences of meeting dogs (e.g. through friends or when out walking) \| \| - None of the above - Other influences (please specify): [free text] \| |

Answer types:

- Multiple choice, one answer
- Multiple choice, multiple answers possible

N.B. Responses were required to all questions presented here, *except* the question asking for the first part of the respondent’s postcode.

**“Choosing My Dog” Interview Guide for pre-arranged interviews with current owners**

N.B. For interviews with *potential* owners, some questions were omitted or amended where appropriate. Text enclosed in square brackets [] refers to guidance and notes for the interviewer.

**Section A – About your dog**

- Can you tell me about your dog?
  - What is their name?
  - How old are they?
  - What breed or type of dog are they?
- How long have you had your dog?
  - How old were they when you got them?
- What is your dog’s daily routine?
  - Who in your household looks after your dog?

**Section B – Deciding to get a dog**

- Is this the first time you’ve owned a dog?
- [If multiple previous dogs it might be useful to make a brief timeline/note all dogs]
- Can you tell me about your previous experiences with dogs?
  - What breeds(s) or types?
  - Were these experiences when you were a child or an adult?
  - How did you get your previous dog(s)
- Who made the decision to get [this dog]?
- How long had you been thinking about getting a dog?
  - How long had you been thinking about getting [this dog]?
  - What prompted you to act when you did?
- Can you describe why you wanted to have a dog?
  - What were the important reasons behind you wanting to have a dog (in general)?
  - How did you imagine having a dog would affect your life (and that of your household)?
  - Did you have any concerns about getting a dog?

**Section C – Before finding your dog**

- You’d decided you wanted a dog: what did you do next?
  - Did you already have a clear idea of what you wanted?
    - Did you know what breed or type of dog you’d like?
    - Did you consider a range of breeds?
    - What about age of dog?
    - Did you have any ideas about where you wanted to get your dog from?
- Did you look for any information or ask anyone for advice before getting your dog?
  - Who did you ask?
  - Where did you look?
  - What information did you want to find?
  - Were you able to find that information?
  - Was there any information you couldn’t find but you would have liked?
  - Approximately how long did you spend looking for information?
    - Was it a long or short process?
- What were important factors when trying to narrow down which breed or type of dog you wanted [if not already covered]?
  - What attracted you to a particular breed or type?
    - Had you had any previous experiences with the breed/type?
    - E.g. family/friends owned this breed/type?
  - What do you like about this particular breed?
- Did you change your mind or have any second thoughts during this process?
  - Was it an easy decision or did you rethink anything?
  - What did you do as a result of any concerns?

**Section D – Finding your dog**

- Where did you get [your dog] from?
  - Was it a breeder/rehoming centre, etc.
    - Did this matter to you at the time?
      - Why/why not?
  - Why did you choose this source?
  - How did you find this source?
  - Was this the only place you looked?
- [lf no, probe more about other dogs they may have enquired about, e.g.]
  - - Did you see any other dogs you liked?
    - Did you speaker to the seller/rescue?
    - What happened?
    - Why did you decide against that dog?
  - What other sources did you consider?
    - What process did you go through to look for possible sources?
    - How did you know where to look?
- When was the first time you met [your dog]?
  - What was the place like?
  - What happened?
    - If [your dog] was a puppy, did you meet your dog’s mother/siblings?
  - What questions did you have (if any)?
  - Were there any negatives about that experience?
    - Was there anything that made you have second thoughts or want to ask more questions?
  - How did you feel?
- How did you decide on [your dog] [if not already covered]?
  - Did you already have a clear idea of what you wanted?
- How many times did you meet your dog before you took them home?
  - Over what period of time?
- What did your dog come home with?
  - Was there any after care offered?
  - E.g. Would any support be offered if there were any issues?
- Who was involved in the process?
  - E.g. household member/family?
    - [If not already know, ask about their previous experiences with dogs]
  - How were they involved in the process?
  - Was it a long or short process?
  - Was it easy or difficult?
  - How did you feel?
- Was it an easy decision or did you rethink anything?
  - Did you change your mind during this process?
  - What did you do as a result of any concerns
- How long overall do you think it took you to get [your dog]?

**Section E – After acquiring your dog**

- How does [your dog] compare to the dog you thought you’d have, before you got them?
  - E.g. Is the breed or type of dog what you imagined yourself with?
- What did you imagine life with [your dog] would be like, before you got them?
  - Has it lived up to those expectations?
  - What changes has having a dog made to your life?
  - What have you most enjoyed?
  - Is there anything that’s been different to what you expected?
- Would you recommend this breed or type of dog to potential owners?
  - Why/why not?
- Would you recommend the source you acquired your dog from to potential owners?
  - Why/why not?
- If you were looking for another dog, where would you look now?
- In hindsight, do you feel that you spent enough time making the decision to get a dog?
  - Did you have all the information you needed?
- What advice would you give to other potential dog owners?

**Ending** (brief summary of main points)

- Is there anything else we should have talked about but didn’t – related to getting a dog?
- Have we missed anything that you think is important?
- Thank you so much for your time and sharing your experiences with us. This has been really interesting and is very important for our work. Thank you!

**Table 5. Themes from qualitative data related to reasoning behind dog acquisition**

| **Overarching Themes** | **Themes** | **Definition** | **Sub-Themes** | **Example Quotations** |
| --- | --- | --- | --- | --- |
| **1. Self-Related Motivation** | **1a.Valued Aspects of Human-Dog Relationships** | Participant cites human–dog connections and the emotional relationship that results from this | Companionship | “*…we got him because I need company.”* (Interview – A1KH05C203) |
|  |  |  | Friendship | “*To have a new loyal best friend*.” (Survey FT – Potential owner – 7123) |
|  |  |  | Love or affection | “*There’s no love like having a dog at all. There is no animal that loves you in the same way as a dog*.” (Interview – B1RM1106) |
|  |  |  | Opportunity to nurture | “*Having a dog is having someone to love and look after*.” (Survey FT – Potential owner 8210) |
|  |  |  | Bond | “*I love the bond u [sic] build*.” (Survey FT – Potential owner – 3494) |
|  |  |  | Reciprocity | “*I have always had a dog in my life and I know how much love a dog can bring to a home and how much I have to give to a dog*.” (Survey FT – Current owner – 2190) |
|  |  |  | Interactions | “*To walk through the door after work to a wagging tail and lots of kisses*.” (Survey FT – potential owner – 417) |
|  | **1b.Benefits to Human Health and Wellbeing** | Participant perceives dogs, or dog ownership, to confer benefits on human health and wellbeing | Mood lifter | “*Just every time we’ve walked past people with dogs, I just look at them and go “I want a dog, I want a dog.” They bring happiness don’t they*.” (Interview - B1RM0403) |
|  |  |  | Improve mental health | “*I struggle with mental health issues and depression so I’d rely on a dog to be the reason I want to get up on a morning because if I had a dog they’d be relying on me to look after them and keep them healthy – then it may make me think about how I care about myself*.” (Survey FT - Current and potential owner – 4511) |
|  |  |  | Meaning or purpose | “*I’ve previously had a rabbit, and when he passed away I was lonely and felt a bit purposeless*.” (Survey FT – Current owner - 1927) |
|  |  |  | Emotional support | “*Emotional support for depression*.” (Survey FT – Potential owner - ) |
|  |  |  | Distraction or focus | “*It was definitely the right time for us, because having a dog would provide a bit of support and a bit of distraction*.” (Interview - B1RM0601) |
|  |  |  | Calming support | “*My youngest son was diagnosed with autism, so we got him for him (…) to calm him at night times when he goes to bed*.” (Interview - A1KH08C409) |
|  |  |  | Sense of safety | “*I don’t like to walk solitary. I mean, I was doing a bit but I encountered a few people on the cliffs that felt a bit dodgy. I mean [dog name] hasn’t got a nasty bone in his body, I’ve never seen anything aggressive, he’s never bared his teeth but it’s just that thing, you’ve got a dog with you and you feel safe*.” (Interview - A1KH10C201) |
|  |  |  | Motivation | “*Extra motivation to get out and exercise several times a day*.” (Survey FT – Potential owner – 2603) |
|  |  |  | Conduit for social connection | “*To get me out the house meeting new people*.” (Survey FT – Potential owner – 8642) |
|  |  |  | Exercise/fitness | *“…to improve my own level of fitness*.” (Survey FT – Potential owner – 8888) |
|  | **1c.Dogs Enrich Owner’s Lives** | Participant reports dogs to enhance owner’s lives | Fun | "*Dogs bring so much fun into your life*." (Survey FT – Current owner – 2889) |
|  |  |  | Enjoyment | *“…to make our lives more enjoyable*.” (Survey FT – Current owner – 2074) |
|  | **1d.Dogs Mediate Owner’s Self-Identity** | Participant believes dogs help to form or manage an owner’s identity | Dog- or animal-person or lover | “*I suppose we’re very much animal-people. We had two cats for seven and eight years, respectively. (…) We don’t plan on having kids, so it’s pets for us. Both of us just love dogs. (…) It just feels right to have a dog*.” (Interview - B2RM0301) |
|  |  |  | Integral to sense of self | “*I wasn’t me without a dog and I felt like I had lost part of myself…It’s just me and how I am*.” (Survey FT – Current owner – 243) |
|  |  |  | Impression management | “*If I walked through a park, as a male in my mid-50s, you’ve got funny looks, if you’ve got a dog, no problem. Quite seriously. You feel it, if there’s kids nearby, you can actually feel the vibe from the mothers looking at this guy in their mid-50s walking through the park, near the play park. If you’ve got a dog there’s absolutely no problem*.” (Interview - A1KH08C504) |
|  | **1e.Desire to Participate in Lifestyle Associated with Dog Ownership** | Participant has lifestyle aspirations related to dog ownership | Walks | “*I wanted a dog to go on walks with*.” (Survey FT – Current owner – 609) |
|  |  |  | Training | "*The enjoyment of taking them to training classes and achieving good grades and passes in the various tests together, their progression in life fills you with pride and joy*." (Survey FT - Current owner - 1808) |
|  |  |  | Spending time outside | “*They gice [sic] you a reason to get out there and enjoy the countryside in all weathers*.” (Survey FT – Potential owner – 214).  “*[Dogs] encourage an active and outdoors lifestyle (which my partner and I love)*” (Survey FT – Potential owner – 9292) |
|  |  |  | Dog-related activities | “*A dog more able to work with me in various dog sports after having to retire previous dog due to old age*.” (Survey FT – Current owner – 2638). |
|  | **1f.Dogs as Family Members** | Participant considers dogs as family members | Dogs as part of the family | “*To become part of our family*.” (Survey FT – Current owner – 1369) |
|  |  |  | Expand or complete the family | “*To make our family complete*.” (Survey FT – Current owner – 141) |
|  |  |  | Alternative to children | “*I’m not a person that ever wants children, so I think I’ve replaced children with dogs. I think it’s the same as if you’re a mother that’s broody*.” (Interview - A1KH10C303) |
|  | **1g.Functional Roles Performed by Dogs** | Participant reports a desire for dogs to perform functional roles | Working | “*Brought [sic] for working sheep*.” (Survey FT – Current owner – 2749) |
|  |  |  | Protection | *“…because it’s just me on my own here I’d like a dog that’s big and could be a big, burly – I don’t mean a guard dog – a family pet but one that would have a big booming bark and would make people think twice about breaking in*.” (Interview - B1RM1201) |
|  |  |  | Therapy | “*I would also like to have a dog that I can develop into becoming a PAT dog and a Therapy Dog to help me with my mental health and physical limitations*.” (Survey FT – Potential owner – 8525) |
|  | **1h.Owner’s Ability to Care for a Dog** | Participant’s perceived capacity to care for a dog affects their acquisition decision-making | Able to offer a good home or life | “…*And we felt we could offer a good and loving home*.” (Survey FT – Current owner - 2627) |
|  |  |  | Resources owner has | “*I have always wanted my own dog and now I live on my own in a 3 bed and am at home most of the time so have plenty of time and space to give a dog a good home*.” (Survey FT – Potential owner – 7528) |
|  | **1h.The Right Time** | Participant considers the timing of dog acquisition with respect to their personal biography | Life transitions | “*The time is right as we moved house and I no longer work*.” (Survey FT – Potential owner – 6418)  “*When I bought this flat and when I suddenly started working from home all the stars aligned*.” (Interview - A1KH08C302)  “*We decided to get our very first dog together as soon as we bought our first house*.” (Survey FT – Current owner – 176) |
|  |  |  | Loss/ageing of other pets | “*We had just lost our boy dogs (…) our house was so empty and void of doggy boneos bits and hairs on the carpet. There was not (sic) one to talk to, to care for, to walk with, and it broke our hearts.”* (Survey FT - Current owner - 3340) |
|  |  |  | Long-held desire | “*I have wanted a dog for sometime, however as I live alone and work long hours it’s not good for a dog. I’m due to retire next year so will be looking for my forever friend*.” (Survey FT – Potential owner – 5390) |
|  | **1i.Owner’s History with Dogs** | Participant reflects on their previous experiences with dogs as an influence in their desire to get a dog | Always been around dogs | “*When you’ve had dogs for 20 years it’s, yeah, it’s quite difficult coming in the house not having a dog here*.” (Interview - B1KH1102) |
|  |  |  | Dogs during childhood | “*I always wanted a dog as I grew up around them*.” (Survey FT – Current owner - 526) |
|  |  |  | Friends/family with dogs | “*My in-laws recently got a rescue dog and we loved her so much it motivated us to get a dog*.” (Survey FT – Current owner – 2927) |
|  |  |  | Work with dogs | *“Working in canine hydrotherapy pool last year for a few hours week which gave me my dog ‘fix’ when the pool relocated it was time to bring a forever dog home*.” (Survey FT – Current owner – 4451)  “*Experiences* *working in animal rescue and dog walking as well as dogs I have known and seeing the need for homes for rescue/older dogs*.” (Survey FT – Potential owner – 4221) |
|  |  |  | Prior relationship with acquired dog | “*I fostered him and couldn’t let him go, didn’t set out to get another dog*.” (Survey FT – Current owner – 2008) |
| **2.Social-Based Motivation** | **2a.Pro-Social: Dog-Related** | Participant reports that their desire to get a dog is/was motivated by the wish to benefit another dog | To help a dog in need | “*I feel like there’s a lot of lonely dogs which we can help.*” (Interview - A1KH08C305) |
|  |  |  | Companionship for another dog | “*We had lost another dog, and our surviving dog was extremely lonely and needed a new companion*.” (Survey FT – Current owner – 2502) |
|  |  |  | Behavioural or emotional support for another dog | “*We already had a very timid rescue dog who needed a more confident dog to help him*.” (Survey FT – Current owner – 3546) |
|  |  |  | The dog’s perspective | “*We feel now maybe that it would be nice for [dog name] to have someone else, another dog with him in the home when we’re not there, maybe would make him a bit happier*.” (Interview - B1RM1203) |
|  | **2b.Pro-Social: Human-Related** | Participant reports that their desire to get a dog is/was motivated by the wish to benefit another person | For a child | “*We aren’t able to have anymore children but wanted an addition that would be able to play and run around with our son*.” (Survey FT – Current owner - 190) |
|  |  |  | For the family | “*To help bring the family together.*” (Survey FT – Potential owner – 4146) |
|  |  |  | To give a relative (not child) access to a dog | “*Mother recently lost her dog and not fit enough to regime [sic] herself. Discussed her dog sitting for us when needed.*” (Survey FT – Current owner – 1789) |
|  | **2.c.Influenced by Social Network** | Participant reports their decision-making around dog acquisition as being influenced by other people | Family or joint decision | “*My son who was 12 at the time asked for a dog. However the whole family came to an agreement*.” (Survey FT – Current owner - 3065) |
|  |  |  | Suggested by someone else | “*I was living on the other side of the country from friends and family and on top of isolation I was diagnosed with depression. Friends and family suggested getting a dog would give me purpose and companionship*.” (Survey FT – Current owner - 2876) |
| **3.Dog-Related Positive Affect-Based Motivation** | **3a.Positive Feelings Towards Dogs or Animals** | Participant describes positive beliefs or attitudes about or towards dogs or animals in general | Love of dogs or animals | “*We love dogs. Very simple really*.” (Survey FT – Current owner – 2037) |
|  |  |  | Love of a particular breed | “*You don’t see many pure bred poodles so I needed my own to get my fix!*” (Survey FT – Current owner – 7524) |
|  |  |  | Appreciation of dogs’ innate qualities | “*I love dogs, they are faithful, loyal companions*.” (Survey FT – Potential owner – 9032) |
|  |  |  | Dogs as unique or distinct from other pets | “…*having a dog is different [to a cat], it does enrich in different ways, because you take them out for walks, you meet other dog people*.” (Interview B1RM1201) |
|  |  |  | Fond previous ownership experiences | “*The companionship and unconditional love is something I always cherished with my previous dog*.” (Survey FT – Potential owner – 6169) |
